# An Assessment on the Awareness of Diabetic Retinopathy Among Participants Attending the Diabetes Awareness Camp in Saudi Arabia

**DOI:** 10.7759/cureus.31031

**Published:** 2022-11-02

**Authors:** Mustafa Abdu, Kareem Allinjawi, Huda M Almabadi

**Affiliations:** 1 Optometry, College of Applied Medical Science, University of Jeddah, Jeddah, SAU

**Keywords:** eye check, community, awareness, diabetic retinopathy, diabetes

## Abstract

Aim

The objective of this study is to assess the level of awareness of the effect of diabetes and diabetic retinopathy (DR) on the eye among a sample of the Jeddah community.

Methods

A cross-sectional study was conducted among those attending a diabetes awareness camp in Jeddah, Saudi Arabia, in November 2021. Participants were asked to answer questions in a structured questionnaire that was already used in a previous study. Responses were analyzed using Statistical Package for the Social Sciences (SPSS) version 25 (IBM SPSS Statistics, Armonk, NY, USA).

Results

A total of 352 participants were included in this study, 184 (52.3%) of them were females. Of the participants, only 74 (21%) had diabetes mellitus (DM). The vast majority (94%) of the participants believed that diabetes could affect the eyes, and 94.3% believed that maintaining the level of blood sugar could maintain the eye and the level of vision. Moreover, 77.3% were aware that diabetes could lead to visual impairment and blindness. Around one-third of the total participants and less than half of the diabetic group were found familiar with DR. Although 96% of diabetic participants reported the need for diabetics to get their eyes checked annually, only 70% did so. Lack of awareness of the effect of diabetes on the retina was the main barrier preventing diabetic groups from getting their eye checked.

Conclusion

Despite the good level of awareness among the community and diabetics about diabetes and its effect on the eyes, there is less awareness that DR is one of the most dangerous complications that lead to visual impairments. These findings assure the importance to raise awareness of DR among the community and diabetics and increase awareness of the importance of annual eye examinations.

## Introduction

Diabetes mellitus (DM) is a group of metabolic disorders characterized by hyperglycemia resulting from defects in insulin secretion, insulin action, or both. However, uncontrolled diabetes can lead to several health conditions that include vascular diseases, kidney failure, heart disease, and blindness [1]. DM is considered a major public health concern with an estimation of half a billion people living with DM worldwide, and the number is expected to rise and affect 25% of the world’s total population by 2030 [2]. According to World Health Organization (WHO) reports, Saudi Arabia ranked as the second highest country in the Middle East and the seventh in the world for the rates of diabetes; however, it is estimated that seven million of the population are diabetic [3-5].

Like many systemic diseases, patients with DM often develop several ophthalmic complications, such as glaucoma, cataract, corneal disorders, iris neovascularization, and neuropathies. The most common and potentially most blinding among these complications is diabetic retinopathy (DR) [6,7], which is the cause of blindness in approximately 2.5 million of the estimated 50 million blind people around the world [8,9]. However, the main problem with DR is that the disease could be asymptomatic until there is significant vision loss. Therefore, early detection plays an important role in preventing irreversible blindness resulting from the disease [10,11]. DR is a disease of the retina that affects both type I and type II DM and is characterized by cellular damage and adaptive vascular changes resulting from compromising oxygen and nutrient delivery to the retina [11,12].

Global awareness levels of DM patients toward DR are variable [13-17]. However, in Saudi Arabia, few studies were conducted to evaluate the level of awareness of the effect of diabetes on the eye and especially on the retina. Although a large proportion of diabetic patients in Saudi Arabia are aware of the effect of the disease, knowledge of its risk factors and prevention is little or poor [18,19].

Community awareness of the danger of DM and DR is the most important factor in preventing diabetics from vision impairment and loss. Although several studies screened the awareness level among diabetic patients in Saudi Arabia, there is no previous report that evaluated the awareness of non-diabetic society. However, results from the current study are expected to highlight the level of awareness of the effect of diabetes on the eye in this segment of society.

## Materials and methods

This cross-sectional study was conducted on November 2021 at Red Sea Mall located in Jeddah, Saudi Arabia, during the diabetes awareness camp organized by the Faculty of Applied Medical Science, University of Jeddah. All visitors who were 18 years or older who visited the campsite were invited to participate, and universal sampling was used in this study. The study was approved by the Institutional Review Board (IRB) of the University of Jeddah (UJ-REC-078), and the study was conducted according to the principles of the Declaration of Helsinki. Informed consent was obtained from the participants before enrollment in the study.

The questionnaire

Participants were asked to answer questions in a structured questionnaire that was already used in a previous study [20]. Prior to the use of the questionnaire, permission was taken from the original author, and the questions were subjected to validation through a pilot test and back-to-back translation. The questions were designed in an online version through Google Forms, delivered in Arabic and English languages, and administered to participants by the researchers who conducted the study.

The questionnaire included 17 questions divided into four sections. The first part of the questionnaire included questions about sociodemographic data that includes age, gender, education level, diabetes existence, disease duration, and diabetes type. The second part included questions assessing the participant’s knowledge about the association between diabetes and vision. The third part included questions assessing the participant’s awareness of DR and the importance of annual eye checking for diabetic patients. The last part included questions that highlighted the barriers to accessing eye services.

Data analysis

Data were analyzed using Statistical Package for the Social Sciences (SPSS) version 25 (IBM SPSS Statistics, Armonk, NY, USA). A descriptive analysis was conducted to generate summary tables. Frequency analysis was used for categorical variables, and mean and standard deviation (SD) were used for numerical variables.

## Results

Demographic profile of participants

A total of 352 participants were interviewed in this study, out of which 168 (47.7%) were males and 184 (52.3%) were females. The mean age of the total participants was 33.22 ± 13.58 (range: 18-72 years), and it was 33.83 ± 13.57 and 32.65 ± 14.27 for males and females, respectively. As regards the level of education, 38 (10.8%) were non-educated, 108 (30.7%) were at high school level of education, and 206 (58.5%) were at university or had a higher level of education.

Among all the participants, only 74 (21%) had diabetes mellitus and 278 (79%) were non-diabetics. Of the 74 diabetic participants, 50 (67.57%) had diabetes mellitus of less than 10 years, 14 (18.92%) had diabetes mellitus between 10 and 20 years, and 10 (13.51%) had the disease for more than 20 years. However, 26 (35.14%) participants reported having type 1 diabetes mellitus, and 48 (64.86%) reported that they had type II diabetes mellitus (Table 1).

**Table 1:** Characteristics of participants SD: standard deviation

Characteristic		Mean ± SD (N (%))
Age	Mean ± SD	33.22 ± 13.58
	Range (years)	18-72
Gender	Males	168 (47.7%)
	Females	184 (52.3%)
Education level	Illiterate	38 (10.8%)
	High school	108 (30.7%)
	University	180 (51.1%)
	Postgraduate	26 (7.4%)
Disease groups	Diabetic	74 (21%)
	Non-diabetic	278 (79%)
Disease duration	Never had diabetes	278 (79%)
	1-5 years	38 (10.8%)
	5-10 years	12 (3.4%)
	10-15 years	12 (3/4%)
	15-20 years	2 (0.6%)
	>20 years	10 (2.8%)
Type of diabetes (n = 74)	Type I	26 (35.14%)
	Type II	48 (64.86%)

Participants’ knowledge about the effect of diabetes on the eye and sight

On questions about the effect of diabetes on the eye and vision, the vast majority (94%) of the total participants believed that diabetes could affect the eyes, and 94.3% believed that maintaining the level of blood sugar could maintain the eye and the level of vision. Moreover, 77.3% reported that diabetes could lead to blindness.

In comparison between diabetic and non-diabetic groups, 97.3% of diabetic and 93.2% of non-diabetic participants reported that diabetes could affect the eyes, while 97.3% and 93.5% of diabetic and non-diabetic groups believed that maintaining the level of blood sugar could preserve the eye and vision level, respectively. Moreover, 86.4% of diabetic and 74.8% of non-diabetic groups believed that diabetes could lead to blindness.

As regards participants’ awareness of diabetic retinopathy (DR), 36.6% (129) of the total participants reported that they knew that DR is one of the severe complications of diabetes, which was 47.3% and 33.8% for diabetic and non-diabetic groups, respectively. When participants were asked if they have DR, 91.2% reported that they were not diagnosed with DR, 6.2% reported they were not sure, and only 2.6% reported that they were already diagnosed with DR. However, 63.5% of the diabetic group reported that they were free of DR, 24.3% were not sure if they had DR, and only nine (12.2%) participants were diagnosed with DR (Table 2).

**Table 2:** Questions assessing participants’ awareness of the effect of diabetes on eye and vision

Question		Non-diabetic (N = 278)	Diabetic (N = 74)	Total (N = 352)
Do you believe that diabetes can affect your eyes?	Yes	259 (93.2%)	72 (97.3%)	331 (94%)
	No	19 (6.8%)	2 (2.7%)	21 (6%)
Do you believe that controlling your blood sugar can help preserve your vision?	Yes	260 (93.5%)	72 (97.3%)	332 (94.3%)
	No	18 (6.5%)	2 (2.7%)	20 (5.7%)
Do you believe that diabetes can lead to blindness?	Yes	208 (74.8%)	64 (86.4%)	272 (77.3%)
	No	70 (25.2%)	10 (13.6%)	80 (22.7%)
Do you know what diabetic retinopathy is?	Yes	94 (33.8%)	35 (47.3%)	129 (36.6%)
	No	184 (66.2%)	39 (52.7%)	223 (63.4%)
Do you have diabetic retinopathy?	Yes	0 (0%)	9 (12.2%)	9 (2.6%)
	No	274 (98.6%)	47 (63.5%)	321 (91.2%)
	Not sure	4 (1.4%)	18 (24.3%)	22 (6.2%)

In a question on the source of information diabetic participants learn about DR, 21.6% reported that they have no idea about DR, 41.9% had their information from their ophthalmologists and doctors, 24.3% from different social media, and 12.2% had their knowledge from family and friends. In another question that asked about the level of awareness of DR (from 0 to 10 scale), it was found that the mean rate of awareness was 4.3 ± 3.5 and 3.1 ± 2.6 for diabetic and non-diabetic participants, respectively.

When participants were asked whether it is important for diabetics to have their eyes checked annually by eye care providers, the majority of the total participants (90.9%) reported yes, which was 89.6% and 95.9% for non-diabetic and diabetic groups, respectively. Unsurprisingly, only 47.2% of the total participants reported that they never got their eyes checked annually. However, 41% of the non-diabetic group and 70.3% of the diabetic group reported that they visit their eye specialists for an annual check. As regards the barriers preventing participants from getting annual screening for their eyes, 55.1% reported a lack of awareness of the effect of diabetes on the retina, 9.7% stated the difficulty in accessing primary eye care services, 12.5% mentioned that the cost is the main barrier, and 22.7% mentioned their fear from discovering that they had DR (Table 3).

**Table 3:** Barriers preventing participants from getting an eye screening

Barrier	Non-diabetic (N = 278)	Diabetic (N = 74)	Total (N = 352)
Lack of awareness of diabetic retinopathy	153 (55%)	41 (55.4%)	194 (55.1%)
Difficulty accessing primary eye services	28 (10.1%)	6 (8.1%)	34 (9.7%)
Cost	37 (13.3%)	7 (9.5%)	44 (12.5%)
Fear of discovering diabetic retinopathy	60 (21.6%)	20 (27%)	80 (22.7%)
Total	278 (100%)	74 (100%)	352 (100%)

## Discussion

Diabetic retinopathy (DR) is one of the leading causes of visual impairment and blindness worldwide. It has been estimated that 285 million people had diabetes mellitus and approximately one-third (34.6%) of them had signs of DR [21,22].

The prevalence of DR among diabetic patients varies from one region to another in Saudi Arabia. The following prevalences have been reported: 33.7% in Riyadh [23], 30% in the eastern area [24], 27.8% in the southern area [25], and 52.3% among diabetic patients who attended Khyber General Hospital in Madinah region [26]. However, the prevalence of blindness due to DR varies accordingly, and it was reported to be 5.7% and 1% in the southern region and Riyadh, respectively [23-25].

Besides the medical deterioration of diabetes, several risk factors were reported to be associated with the increased prevalence of DR among diabetic patients. These factors include the long duration of diabetes, high levels of hemoglobin A1c (HbA1c), high blood pressure, and the level of awareness of the effect of diabetes [27,28]. Therefore, community awareness of the danger of DM and DR is the most important factor in preventing diabetics from vision impairment and blindness.

The current study was designed to assess the level of awareness of the community (including diabetic patients) of the effect of diabetes on the eye, especially DR, and it was found that a high percentage of the study community was aware that diabetes could affect the eyes, and the majority of the participants were also aware that maintaining the level of blood sugar could save the eyes and vision. Moreover, more than two-thirds of the participants were also aware that diabetes could lead to blindness. Interestingly, the level of awareness was analogical between diabetic and non-diabetic participants in the study group.

Comparing the awareness level of diabetic participants of the effect of the disease on the eye with that reported in the literature, it was noticed that the current percentage (97.3%) is close to that reported in Jordan (98.3%) [20], Switzerland (96%) [29], and Syria (93.8%) [14] and slightly higher compared to that found in Ghana (80.2%) [30] and India (74.3%) [31]. In Saudi Arabia, the awareness level is the highest compared to other local studies in Jeddah (92.4%) [32] and Riyadh (91%) [33].

Around two-thirds of the study community were not aware that DR is one of the most serious complications of diabetes, with increased awareness among the diabetic group (47.3%) compared to the non-diabetic group (33.8%). Similar findings were reported in studies among diabetic patients in Ghana (48.8%) [30] and Ethiopia (47.4%) [34] and apparently less compared to that reported in Qassim province in Saudi Arabia (63.5%) [35] and North India (69.5%) [36] as they recruited participants already diagnosed with diabetes.

General practitioners and eye care providers were the main sources of information on diabetes and DR for almost 42% of the diabetic group in the current study. This finding is consistent with studies in Jordan [20] and Pakistan [37], which reported that doctors were the main source of information on diabetes and DR.

Among all of the study community, around 91% reported the importance of diabetics having their eyes annually checked by eye specialists with a slight difference between diabetic and non-diabetic groups. Despite this high level of awareness, the study revealed that around 50% of the community never had a checkup from ophthalmologists, while almost 70% of the diabetic group are already seeing their ophthalmologists annually for an eye examination. The finding among the diabetic group of the current study is the highest compared to that reported earlier in Jordan (38.3%) [20], Sudan (35.5%) [38], and Myanmar (57%) [39] and almost similar to that reported in Syria (63%) [14] and Japan (69.5%) [40]. The low level of awareness recorded in some studies signifies the need for further health education for patients on ocular complications associated with diabetes for better knowledge and better management. Of the diabetic participants in the current study, 55% stated that lack of awareness of DR is the main barrier to not receiving an annual eye check. This finding is comparable with that reported in similar studies by El Khatib et al. [20] in Jordan and Dervan et al. [41] in Japan, which concluded that the main barrier to receiving adequate eye checks for diabetic patients is the lack of awareness and knowledge about the danger of DR.

The limitation of this study is the sampling process, as convenient sampling was used, which results in the inequality between the number of diabetics and non-diabetics and which may have an impact on the results when comparing these groups.

## Conclusions

The current study concluded that despite the good level of awareness among the community and diabetic patients about diabetes and its effect on the eyes, there is less awareness that DR is one of the most dangerous complications that lead to vision impairment. Thus, it is important to increase the awareness of DR among the community and diabetics and increase the awareness of the importance of annual eye examinations.
